# TERRA Expression Levels Do Not Correlate with Telomere Length and Radiation Sensitivity in Human Cancer Cell Lines

**DOI:** 10.3389/fonc.2013.00115

**Published:** 2013-05-10

**Authors:** Alexandra Smirnova, Riccardo Gamba, Lela Khoriauli, Valerio Vitelli, Solomon G. Nergadze, Elena Giulotto

**Affiliations:** ^1^Laboratorio di Biologia Molecolare e Cellulare, Dipartimento di Biologia e Biotecnologie “Lazzaro Spallanzani”, Università di PaviaPavia, Italy

**Keywords:** TERRA, telomere length, radiation sensitivity, cancer cell lines, clonal variation

## Abstract

Mammalian telomeres are transcribed into long non-coding telomeric repeat-containing RNA (TERRA) molecules that seem to play a role in the maintenance of telomere stability. In human cells, CpG-island promoters drive TERRA transcription and are regulated by methylation. It was suggested that the amount of TERRA may be related to telomere length. To test this hypothesis we measured telomere length and TERRA levels in single clones isolated from five human cell lines: HeLa (cervical carcinoma), BRC-230 (breast cancer), AKG and GK2 (gastric cancers), and GM847 (SV40 immortalized skin fibroblasts). However, these two parameters did not correlate with each other. Moreover, cell survival to γ-rays did not show a significant variation among the clones, suggesting that, in this cellular system, the intra-population variability in telomere length and TERRA levels does not influence sensitivity to ionizing radiation. This conclusion was supported by the observation that in a cell line in which telomeres were greatly elongated by the ectopic expression of telomerase, TERRA expression levels and radiation sensitivity were similar to the parental HeLa cell line.

## Introduction

Telomeres are nucleoprotein complexes, located at the ends of linear eukaryotic chromosomes, playing an essential role in the maintenance of chromosome integrity. In mammalian cells, telomeres are composed by extended tracts of (TTAGGG)n DNA repeats and by a protein complex called shelterin. While in somatic cells telomeres shorten at a constant rate at each cell division (Harley et al., 1990), due to the inability of DNA polymerase to complete replication of the lagging strand, in germline and stem cells, telomere length is maintained by the specialized enzyme telomerase (Greider and Blackburn, 1985; Wright et al., 1996). Also in cancer cells telomere length is maintained, usually through telomerase activation (Artandi and DePinho, 2010); however, in about 15% of human cancers, an alternative telomere lengthening (ALT) mechanism operates which does not require telomerase activation. In ALT cells, telomere maintenance is due to a recombination based mechanism (Bryan et al., 1997) causing great intracellular variability of telomere length (Henson et al., 2002).

It is widely accepted that the length of telomeres is determined by the balance between the rate of shortening at each replication cycle and the rate of elongation by telomerase or ALT mechanisms. The regulation and dynamics of this equilibrium are still not fully understood: different factors seem to play a role in sensing telomere length and in modulating telomere elongation (Palm and de Lange, 2008). It has been proposed that, among these factors, telomeric repeat-containing RNA (TERRA) may contribute to telomere stability. TERRA is the product of telomere transcription, performed by RNA polymerase II (Azzalin et al., 2007; Schoeftner and Blasco, 2008). In somatic mammalian cells (Azzalin et al., 2007; Schoeftner and Blasco, 2008) as well as in human oocytes (Reig-Viader et al., 2013), TERRA is mainly localized at telomeres suggesting the existence of post-transcriptional mechanisms retaining these RNA molecules where they are synthesized; moreover, it was shown that TERRA is regulated during the cell cycle, being lowest in late S phase and peaking in early G1 (Porro et al., 2010).

Telomeric repeat-containing RNA molecules are heterogeneous in length and contain not only telomeric repeats but also a portion of the subtelomeric transcripts. In human cells, TERRA is transcribed from CpG-island promoters whose methylation levels influence TERRA expression; such promoters are located on 20 subtelomeres (Nergadze et al., 2009; Farnung et al., 2010). Although these observations suggest that TERRA may play a role, specific functions still remain to be assigned to this non-coding RNA fraction. It was shown that TERRA-like oligonucleotides inhibit human telomerase *in vitro* (Redon et al., 2010) and several *in vivo* studies indicate that TERRA levels may contribute to telomere length control. In cells derived from patients affected by the ICF syndrome (immunodeficiency, centromere instability, and facial abnormalities type I), hypomethylation of the subtelomeric regions is associated with increased TERRA levels and shortened telomeres (Yehezkel et al., 2008). In yeast, the rat1p exonuclease is known to degrade TERRA and rat1p mutant strains, in which TERRA levels are increased, telomeres are shortened (Luke et al., 2008); in addition, TERRA over-expression from an artificially induced promoter seem to cause telomere shortening (Pfeiffer and Lingner, 2012). Altogether, these pieces of evidence suggest that TERRA may promote telomere shortening, however, recent results show that the involvement of TERRA in telomere homeostasis is more complex. The *in vitro* observation that TERRA is an inhibitor of telomerase was not confirmed *in vivo*: in human cancer cells, telomerase-mediated telomere elongation is not affected by TERRA expression (Farnung et al., 2012). It has also been suggested that the relationship between TERRA and telomere length could be mediated by chromatin reorganization at telomeres. In a recent work (Arnoult et al., 2012), TERRA expression from specific telomeres was analyzed in cell lines in which telomeres were elongated by the ectopic expression of telomerase and in a HeLa clone with naturally long telomeres. In these systems, longer telomeres seemed to be associated with lower TERRA levels and the analysis of histone composition suggested that silencing of TERRA expression at longer telomeres could be mediated by the H3K9me3 histone variant.

To elucidate the possible relationship between TERRA levels and telomere length, in the work presented here we measured these parameters in isogenic clonal populations derived from five human cancer cell lines and in a cell line in which telomeres were greatly elongated by the ectopic expression of telomerase. In addition, since previous observations suggested that telomere length may influence the cellular response to ionizing radiation (Wong et al., 2000; McIlrath et al., 2001; Drissi et al., 2011), we analyzed γ-ray sensitivity in clones characterized by different telomere lengths and TERRA expression levels.

## Results and Discussion

The aim of this work was to test whether a correlation exists between telomere length and TERRA expression level and to verify if either of these factors can influence radiosensitivity.

### TERRA level in HeLa cells overexpressing telomerase

**Figure 1:**
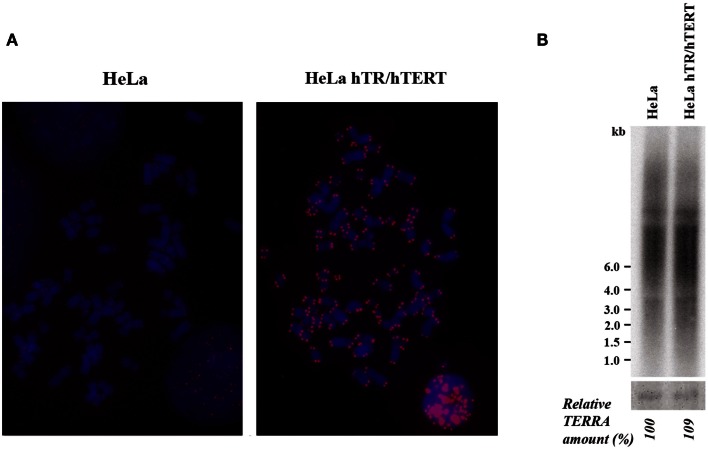
**(A)** Telomere detection in HeLa and hTR/hTERT cells by PNA-FISH. Telomeric signals are in red, chromosomes are stained with DAPI. **(B)** Northern blot analysis of TERRA expression in HeLa and hTR/hTERT cells. Ethidium bromide-stained 18S rRNA bands are shown at the bottom.

We initially analyzed HeLa hTR/hTERT cells, a previously established cell line in which telomeres were greatly elongated by the ectopic expression of both the RNA and of the reverse transcriptase subunits of human telomerase (Cristofari and Lingner, 2006). While in the parental cell line telomeres were on average 7 kb long, in hTR/hTERT cells, telomeres were up to 50 kb long, as determined by Terminal Restriction Fragment (TRF) analysis (Cristofari and Lingner, 2006). In Figure 1A the results of a fluorescent *in situ* hybridization experiment with a telomeric PNA probe are shown: the intense signals at all hTR/hTERT telomeres confirm that the transduced cell line has longer telomeres compared to its parental cell line. We then measured TERRA levels by northern blotting using a telomeric DNA probe (Figure 1B): the intensity of the autoradiographic signals in the hTR/hTERT and in the parental line were similar indicating that the total amount of TERRA was not influenced by telomere elongation. This result is in agreement with a recent finding from Farnung et al. (2012) who showed that, in a hTERT infected HeLa cell line in which telomeres were elongated, no substantial alteration in total TERRA levels was detectable.

### TERRA levels and telomere length in clones isolated from tumor cell lines

Since it was previously shown that in tumor cell lines telomere length can be heterogeneous (Bryan et al., 1998; Savre-Train et al., 2000), we planned to measure TERRA levels and telomere length in a number of clones isolated from different human cell lines in search of a possible correlation between these two parameters.

**Figure 2:**
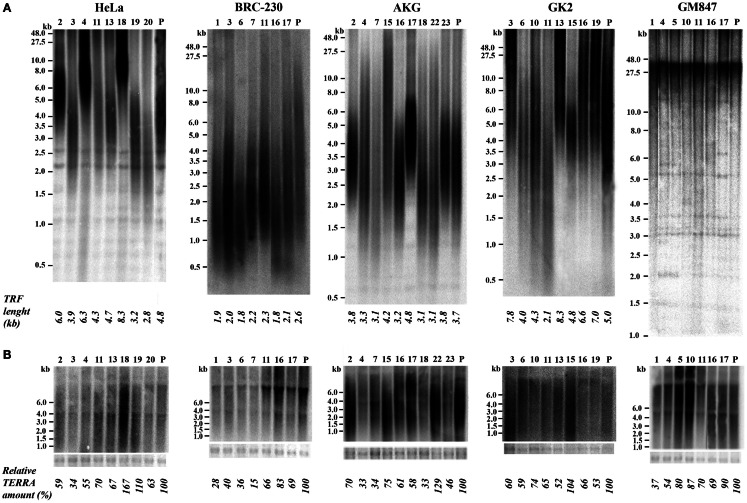
**(A)** Terminal Restriction Fragment analysis by Southern blotting in parental (P) HeLa, BRC-230, AKG, GK2, and GM847 cell lines and in their clones (numbers on top). Average TRF length is shown at the bottom. **(B)** TERRA expression detection by northern blotting in the same parental cell lines and in the clones. Ethidium bromide-stained 18S rRNA bands (bottom) were used as loading control.

We isolated single clones from five human cell lines. Four of these cell lines derive from different types of tumors: HeLa, from cervical carcinoma (Scherer et al., 1953); BRC-230, from primary ductal infiltrating breast carcinoma (Amadori et al., 1993); AKG and GK2, from a gastric cancer pleural and an ascitic effusion, respectively (Bertoni et al., 1998). One cell line, GM847, derives from human skin fibroblasts immortalized by SV40 (Bryan et al., 1995); in this line telomerase is inactive and telomeres are maintained through the ALT mechanism. At least seven clones were isolated from each cell line; the clones were expanded until about 10^8^ cells, RNA and genomic DNA were then extracted. For TRF analysis the DNA was digested with the restriction enzymes *Rsa*I and *Hin*fI, separated by electrophoresis, blotted to nylon membranes and hybridized to a telomeric DNA probe (Bertoni et al., 1994; Azzalin et al., 1997). Mean TRF length was measured using the densitometric method and the equation previously described (Kimura et al., 2010). Total TERRA levels were evaluated by northern blotting followed by densitometric analysis of signal intensities on the autoradiograms. In Figure 2A the results of TRF length analysis of the clones and of their respective parental cell lines is shown. In the eight HeLa clones the heterogeneity in TRF average length was remarkable, ranging from 2.8 to 8.3 kb, while TRF average length of the parental cell lines was approximately in the middle of this range. TRF length variation, comprised between 2.1 and 8.3 kb, was also observed among the clones derived from the GK2 gastric cancer cell line and, to some extent, among those derived from the other gastric cancer line, AKG (3.1–4.8 kb). In the seven clones isolated from the breast cancer cell line BRC-230 and in their parental cell line, TRFs were always rather short being ranging from 1.8 to 2.6 kb. In all the clones derived from the GM847 cell line, most TRFs were longer than 20 kb, however, shorter fragments, distributed over a wide range of sizes, were also detected; in the parental GM847 cell line a similar distribution of TRFs was previously described (Bryan et al., 1995). Great intracellular variation of telomere length is a typical feature of ALT cells (Henson et al., 2002) and seems to be a direct consequence of the recombination events involved in the maintenance of telomeres. The distribution of TRFs in the seven clones and in the GM847 parental line is similar (Figure 2A), however, we cannot exclude that some variation in the high molecular weight fragments may be present but is not detectable using conventional electrophoresis.

**Figure 3:**
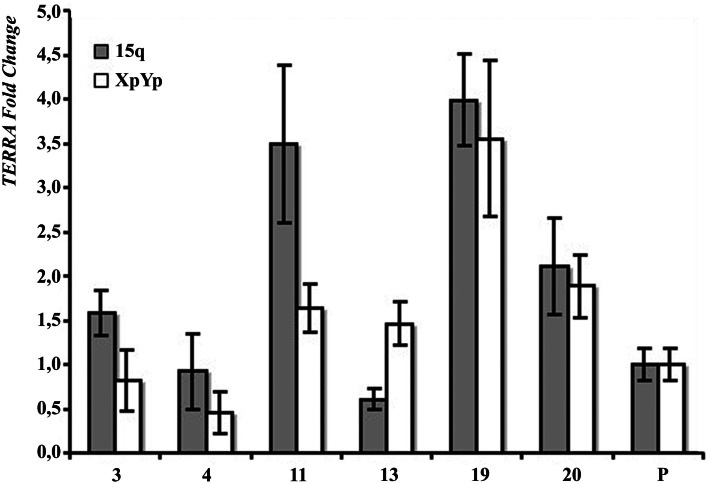
**Subtelomere-specific TERRA levels in HeLa parental line (P) and six clones, determined by qRT-PCR on 15q (gray bars) and XpYp (white bars) subtelomeres**. The values of HeLa parental line is arbitrarily set at 1. Average values from three independent reactions are shown.

In Figure 2B the results of northern blotting experiments performed with total RNA from all the clones and from their respective parental cell lines are shown. TERRA levels were evaluated as ratio between signal intensity in each clone and in its parental cell line; these values showed inter-clonal heterogeneity, ranging from 34 to 167% in HeLa cells, from 15 to 83% in BRC-230, from 33 to 129% in AKG, and from 52 to 104% in GK2 cells (Figure 2B). Also the clones isolated from the ALT cell lines GM847 showed variable TERRA levels, ranging from 37 to 90%. In six of the HeLa clones, TERRA levels from specific chromosome ends were also evaluated by quantitative RT-PCR using primers for the 15q and the Xp/Yp subtelomeres; the amount of TERRA in each clone was normalized with respect to the value of the parental cell line. As shown in Figure 3, TERRA expression from the two subtelomeres follows a similar trend and inter-clonal variability was observed also with this type of analysis: TERRA levels in the different clones ranged from 0.45 to 4 times those of the parental cell line. The comparison between northern blot and qRT-PCR results, indicates that telomere specific expression does not always reflect total TERRA levels; for example in the clones 11 and 13 the total amount of TERRA is very similar (Figure 2B); however, while the expression of the XpYp subtelomere in the two clones is also similar, TERRA levels from the 15q subtelomere are about six times higher in clone 11 compared to clone 13. These results support the hypothesis that the regulation of TERRA expression is telomere specific, as previously suggested (Deng et al., 2012).

**Figure 4:**
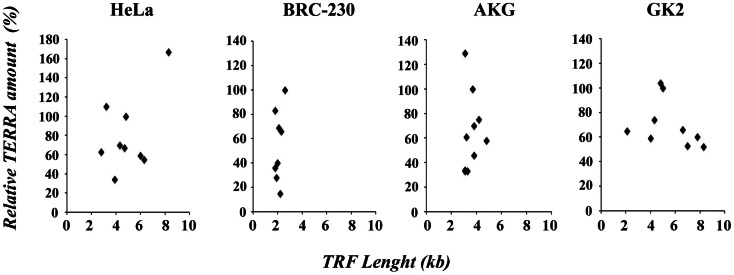
**Scatter plots of TRF length versus relative TERRA amount in HeLa, BRC-230, AKG, and GK2 parental cell lines and in their clones**.

In Figure 4, the values of TRF length and total TERRA levels, are displayed in four scatter plots comprising data of HeLa, AKG, GK2, and BRC-230 clones, respectively. The plots show that the two parameters are independent. These results are in contrast with the previous hypothesis according to which long telomeres should correspond to low TERRA amounts. This hypothesis was initially postulated based on the observation that TERRA-like oligonucleotides inhibit human telomerase *in vitro* (Redon et al., 2010). However, as mentioned above, the effect of TERRA on telomerase activity was not confirmed *in vivo* (Farnung et al., 2012). A negative correlation between telomere length and TERRA level was also observed in ICF patients (Yehezkel et al., 2008) and in cell lines expressing exogenous telomerase or carrying naturally long telomeres (Arnoult et al., 2012).

### Radiation sensitivity

In telomerase deficient mice and in cells derived from them, a clear correlation between telomere shortening and radiosensitivity was demonstrated (Goytisolo et al., 2000; Wong et al., 2000; Latre et al., 2003; Genescà et al., 2006); also in human cells in which telomerase is not expressed, such as primary fibroblasts (Drissi et al., 2011) and lymphocytes (McIlrath et al., 2001), an inverse correlation between telomere length and radiosensitivity was observed; whether this correlation holds true in telomerase positive cells is still a matter of debate. In a previous work, we showed that three telomerase immortalized fibroblast clones, characterized by different TRF length, had the same radiosensitivity (Zongaro et al., 2008), whereas radiosensitive murine lymphoma cells exhibited a sevenfold reduction in telomere length in comparison with their parental radioresistant cells (McIlrath et al., 2001). To gain new insight into this matter and to elucidate whether TERRA expression may be related to radiosensitivity, we evaluated the sensitivity to γ-irradiation in the cellular systems described above.

**Figure 5:**
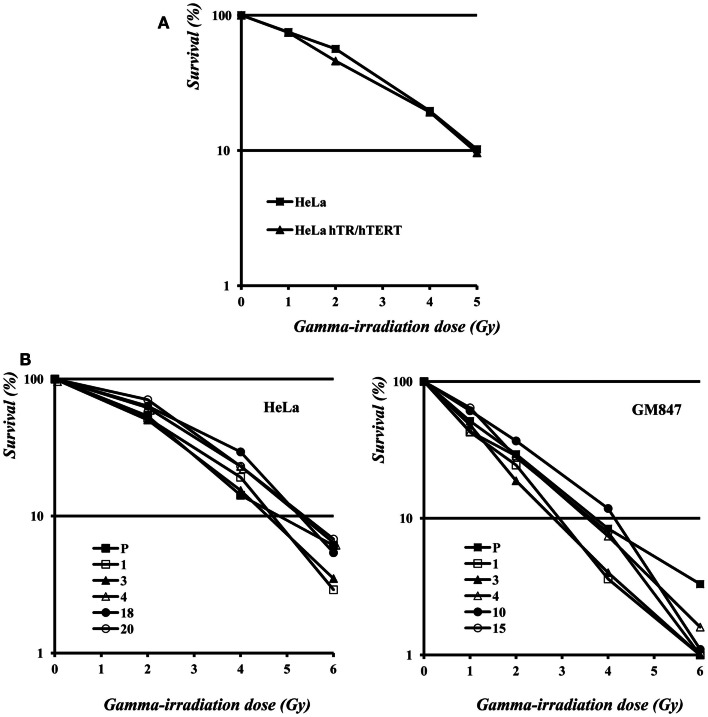
**(A)** Survival to γ-irradiation of HeLa and hTR/hTERT cells. **(B)** Survival to γ-irradiation of HeLa (left) and GM847 (right) cells and of their clones.

As shown in Figure 5A, the survival curve of HeLa hTR/hTERT cells is nearly identical to the one of the HeLa parental cell line. Therefore, in a telomerase positive background, telomere elongation due to telomerase hyper-expression does not make cells more resistant to ionizing radiations. We then measured the survival to γ-irradiation of five HeLa, five GM847 subclones, and their parental cell lines. In the five HeLa derived clones, although telomere length and TERRA expression levels were variable (Figure 2), the sensitivity to ionizing radiation was similar. The survival curves of the GM847 cell line and its five clones are also similar (Figure 5B) suggesting that, in ALT cells as well as in telomerase positive cells, TERRA expression does not influence radiation sensitivity. However, GM847 cells are more sensitive to irradiation (10% survival between 2 and 4 Gy) than HeLa cells (10% survival at 5 Gy); this relative hypersensitivity may be related to the ALT phenotype.

## Conclusion

In conclusion, in the work presented here we did not find any correlation between TRF length and TERRA levels in isogenic cell lines in which these parameters were naturally different. We also observed similar levels of TERRA in cells with extremely elongated telomeres, expressing exogenous telomerase, and in their parental cell line. Moreover, in the cellular systems exploited here, telomere length and TERRA expression are not related to alterations in the sensitivity to ionizing radiations.

## Materials and Methods

### Cell culture

In this work we used six human cell lines: HeLa, human cervical carcinoma; hTR/hTERT, HeLa cells transduced with retroviral vectors encoding hTR and hTERT (Cristofari and Lingner, 2006); BRC-230, cell line established from surgical material of primary ductal infiltrating breast carcinoma (Amadori et al., 1993); AKG and GK2, two gastric cancer cell lines established from a pleural and an ascitic effusion, respectively (Bertoni et al., 1998); GM847, an SV40 immortalized human skin fibroblasts cell line. All cell lines were routinely cultured in 5% CO_2_ at 37°C. HeLa, HeLa hTR/hTERT, and GM847 cell lines were propagated in Dulbecco’s Modified Eagle’s Media (Euroclone), supplemented with 10% Fetal Bovine Serum (Euroclone), 1% l-glutamine (Sigma), 1% Penicillin–Streptomycin (Sigma) and 1% non-essential amino acids (Euroclone). For BRC-230, AKG and GK2 cell lines the culture medium was composed of a 1:1 mixture of Dulbecco’s Modified Eagle’s Medium (Euroclone) and of HAM’S Nutrient Mixture F12 (Euroclone), supplemented with 10% Fetal Bovine Serum (Euroclone), 1% l-glutamine (Sigma), 1% Penicillin–Streptomycin (Sigma), and insulin 10 μg/ml (Sigma).

### RNA extraction and northern blotting

RNA extraction from whole cells was performed using TRIzol reagent (Invitrogen) according to manufacture’s protocol. To eliminate any DNA contaminations, the RNA was treated twice with RNase-free DNase I (Promega), and then purified with the RNA Clean and Concentration kit (ZYMO Research). For northern blots, 10 μg of RNA were electrophoresed in 1.2% formaldehyde agarose gels and blotted to nylon membranes (Amersham HybondTM-N, GE Healthcare). The membranes were hybridized for 18 h in Church buffer containing ^32^P-α[dCTP]-labeled probes at 58°C. The strand-specific telomeric probe used to detect total TERRA was described previously (Azzalin et al., 2007). After hybridization, the membranes were washed twice with a 2× SSC, 0.5% SDS solution for 15 min at 58°C and once with a 0.2× SSC, 0.5% SDS solution for 30 min at 58°C. Radioactive signals were detected using a phosphorimager (Cyclone, Packard).

### Reverse transcription and qPCR

Total RNA purified as described above was reverse transcribed using the RevertAid Premium First Strand cDNA Synthesis Kit (Fermentas) using the primers: (CCCTAA)_5_ at a final concentration of 0.4 μM for TERRA molecules and GGAACTCGAGTTTGCGTGTCATCCTTGCGC at a final concentration of 0.04 μM for U6 snRNA, used as a control.

We PCR amplified 2 μl of reverse transcription reaction using GoTaq qPCR Master Mix (Promega), in a final volume of 25 μl. To amplify TERRA molecules, we used the following pairs of primers: CAGCGAGATTCTCCCAAGCTAAG and AACCCTAACCACATGAGCAACG for TERRA derived from the 15q subtelomere, GCAAAGAGTGAAAGAACGAAGCTT and CCCTCTGAAAGTGGACCAATCA for TERRA derived from the XpYp subtelomere. As a normalization control, we amplified U6 snRNA using GGAATCTAGAACATATACTAAAATTGGAAC and GGAACTCGAGTTTGCGTGTCATCCTTGCGC as primers. The following PCR profile was used: 5 min at 95°C, 45 cycles at 95°C for 10 s and 60°C for 30 s. Data were analyzed with the Opticon^®^ Monitor 3 software.

### DNA extraction and southern blotting

Genomic DNA was extracted with phenol/chloroform standard method and purified with Proteinase K and Ribonuclease A (Sigma). For TRF length measurement, 10 μg of high molecular weight genomic DNA were digested with the *Rsa*I and *Hin*fI restriction enzymes (Fermentas). After electrophoresis in 0.6% agarose gel, DNA was denatured and transferred to nylon membranes (Amersham HybondTM-N, GE Healthcare). The membranes were hybridized at 64°C for 18 h in Church buffer containing a ^32^P-α[dCTP]-labeled probe generated by random primer labeling of a mixture of 1–5 Kb long telomeric DNA fragments, prepared as previously described (Bertoni et al., 1994; Azzalin et al., 1997). After hybridization, the membranes were washed twice in 2× SSC, 0.5% SDS for 15 min at 64°C and once in 0.2× SSC, 0.5% SDS for 30 min at 64°C. Radioactive signals were detected using a phosphorimager (Cyclone, Packard).

### Data analysis

We measured TERRA amounts quantifying northern blotting signals and 18S rRNA ethidium bromide-stained bands with the software ImageJ. TERRA signals were normalized against the intensity of 18S rRNA bands. For every cell line the parental line were arbitrarily set at 100%. Average telomere length was determined by comparing the molecular weight of the telomeric signal with a calibration curve based on the molecular weight marker, as previously described (Kimura et al., 2010).

### PNA-FISH

PNA-FISH was carried out as previously described (Ruiz-Herrera et al., 2011). Briefly, metaphase spreads were denatured at 72°C for 3 min and then hybridized with a cy3-conjugated PNA (CCCTAA)_5_ probe (Panagene) in 10 mM TRIS-HCl pH 7.2, 70% formamide and 0.5% blocking reagent (Roche). Post-hybridization washes were carried out twice for 15 min in 70% formamide, 10 mM TRIS-HCl, 0.1% BSA at pH 7.5, and then three times for 5 min in TBS buffer (0.1 M TRIS-HCl, 0.15 M NaCl, 0.08% Tween 20 pH 7.5). Chromosomes were dehydrated in ethanol, air dried and counterstained with DAPI 0.2 mg/ml. Image were acquired using a ZEISS Axioplan fluorescence microscope equipped with a Photometric CCD camera and processed with IP-Lab software.

### Sensitivity to irradiation

Exponentially growing cells were trypsinized, resuspended in complete medium and irradiated. Irradiation was carried out using a ^60^Co-ray source at a dose rate of 1.3 Gy/min. After irradiation, the cells were diluted and plated in 100-mm dishes (500 cells/plate). After 10 days, colonies were fixed, stained and the number of colonies with more than 50 cells was counted.

## Conflict of Interest Statement

The authors declare that the research was conducted in the absence of any commercial or financial relationships that could be construed as a potential conflict of interest.
